# Post endodontic pain following single-visit root canal preparation with rotary vs reciprocating instruments: a meta-analysis of randomized clinical trials

**DOI:** 10.1186/s12903-017-0355-8

**Published:** 2017-05-25

**Authors:** Xiao-Mei Hou, Zheng Su, Ben-Xiang Hou

**Affiliations:** 1 The Second Dental Center, Peking University School and Hospital of Stomatology, Beijing, China; 2 Department of Endodontics, Capital Medical University School of Stomatology, No. 4 Tian Tan Xi Li, Dong Cheng, Beijing, 100050 China

**Keywords:** Endodontic therapy, Post-endodontic pain, Rotary, Reciprocal, Endodontic instruments

## Abstract

**Background:**

In endodontic therapy, continuous rotary instrumentation reduced debris compared to reciprocal instrumentation, which might affect the incidence of post-endodontic pain (PP). The aim of our study was to assess whether PP incidence and levels were influenced by the choice of rotary or reciprocal instruments.

**Methods:**

In this meta-analysis the Pubmed and EM databases were searched for prospective clinical randomized trials published before April 20, 2016, using combinations of the keywords: root canal preparation/instrumentation/treatment/therapy; post-operative/endodontic pain; reciprocal and rotary instruments.

**Results:**

Three studies were included, involving a total of 1,317 patients, 659 treated with reciprocating instruments and 658 treated with rotary instruments. PP was reported in 139 patients in the reciprocating group and 172 in the rotary group. The PP incidence odds ratio was 1.27 with 95% confidence interval (CI) (0.25, 6.52) favoring rotary instruments. The mild, moderate and severe PP levels odds ratios were 0.31 (0.11, 0.84), 2.24 (0.66, 7.59) and 11.71 (0.63, 218.15), respectively. No evidence of publication bias was found.

**Conclusions:**

Rotary instrument choice in endodontic therapy is associated with a lower incidence of PP than reciprocating instruments, while reciprocating instruments are associated with less mild PP incidence.

## Background

Endodontic treatment includes preparation and sealing of the root canals, followed by the healing of periradicular tissues [1]. Post-endodontic pain (PP) can occur within a few hours or a few days after endodontic treatment [2]. The incidence of PP is reported to range from 13.15 to 64.7% [3–5], and varies between reports according to study type (prospective or retrospective), selection of patients, time of tooth pulp and apical periodontitis diagnosis, experience and qualification of the dentist, and the time point when pain is recorded [5–8]. The Visual Analogue Scale (VAS) was widely used to evaluate the PP [9], which is represented as a continuous line with numbers from 1 to 100 marked along the line, reflecting pain intensity. PP intensity typically ranges from 5 to 44 points within 72 h after endodontic treatment, and responds well to non-steroidal anti-inflammatory drugs and acetaminophen [10].

Despite an abundance of studies on the topic, the mechanism of PP remains unclear. PP is usually attributed to a complex multifactorial process [11] influenced by sex (PP is reported more often by females than males), pulpal and periradicular status, tooth type, sinus tracts, preoperative pain, systemic steroid therapy for other medical reasons, preoperative swelling and number of treatment visits [4, 12–15]. PP could also occur as a result of inadequate instrumentation, extrusion of irrigation solutions, extrusion of intra-canal dressing, traumatic occlusion, missed canals, preoperative pain, periapical pathosis and extrusion of apical debris. Furthermore, instrument choice might also play an important role. The apical extrusion of infected debris during chemo-mechanical instrumentation of root canals might exacerbate the inflammatory response and cause periradicular inflammation [16]. The shaping procedure itself may promoteapical extrusion of debris [17]. Factors such as the irrigation protocol [18], final apical size [19], time spent on root canal instrumentation [20], technique employed [21] and instrument design [22] can also affect the extrusion of debris.

Nickel-titanium (NiTi) rotary files have been shown to extrude less debris than stainless steel hand files [23]. Recently, more rotary and reciprocal NiTi instruments have been introduced. It was reported that both single-file reciprocating systems (ie, Wave One and Reciproc instruments) and continuous rotary systems (ie, ProTaper and M two instruments) achieved similar effectiveness regarding reducing endotoxins and cultivable bacteria from primarily infected root canals [24]. However, continuous rotary instrumentation provides a passageway for removal of debris from the root canal, thus reducing apical extrusion of debris, and reducing the severity of post-operative pain [25] when compared to reciprocal instrumentation [26]. However, in a recent clinical randomized trial including 624 patients, the use of reciprocal instrumentation was associated with less postoperative pain than rotary instrumentation [27]. In this meta-analysis we sound to conclusively review the influence of choice of rotary or reciprocal instruments on the incidence of PP in clinical randomized trials.

The primary aim of the study was to investigate whether PP incidence following single visit of root canal preparation evaluated by VAS was similar following procedures using rotary and reciprocating instruments. The secondary outcome was to investigate whether subgroup of the PP levels was similar or not.

## Methods

To evaluate post endodontic pain incidence and levels following single-visit root canal preparation evaluated by VAS with rotary vs reciprocating instruments in randomized controlled clinical trials, articles describing evaluation of PP using VAS were identified by searching MEDLINE and EMBASE using the following key words: root canal preparation; root canal instrumentation; root canal treatment; root canal therapy; postoperative pain/post-endodontic pain; reciprocal and rotary instruments. Only prospective randomized clinical trials comparing PP following root canal preparation using reciprocal and rotary instruments, published before April 20, 2016 in English were included. We excluded reviews; case reports; abstracts; studies comparing different rotary instruments; technology introductions; studies that did not report the incidence of PP by the mean of VAS score.

### Data extraction

From the selected studies, the following criteria were extracted: authors, sample size, randomization, type of post-operative pain evaluation, study period, methodology and main outcomes. Data on the use of different rotary instruments were combined. All reported pain levels (mild, moderate, severe) were combined to calculate the incidence of PP. Analgesic dose were categorized as follows: 1 tablet = mild; 2 tablets = moderate; 3 tablets = severe if necessary [28].

### Assessment of risk of bias

Risk of bias was independently evaluated by two reviewers in accordance with the Cochrane risk of bias tool. Disagreements were solved by discussion. The quality evaluation was assessed according to random sequence generation, blinding allocation, participants, personnel and outcome assessment, incomplete outcome, selective reporting and other sources of bias.

### Statistical methods

Trial outcome data was pooled into odds ratio (OR) for dichotomous outcomes using Rev Man 5.3 software. Heterogeneity was estimated using the *I* ^*2*^ test and *P* value. The heterogeneity of data was predefined as *P* < 0.1and *I* ^*2*^ > 50%. Where substantial heterogeneity (*P* < 0.1and *I* ^*2*^ > 50%) were observed, a random-effects model was used. Otherwise, the fixed-effects model was used. Publication bias was evaluated using funnel plot.

## Results

**Fig. 1:**
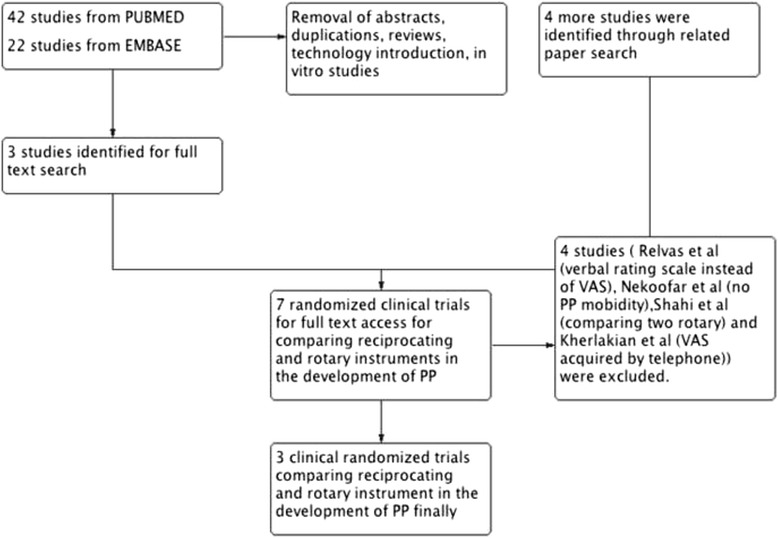
Flow chart of the included studies: there were 66 studies searched and 3 studies were finally included

Forty-two studies were identified by searching PUBMED and 22 studies were identified by searching EMBASE. After exclusion of abstracts, reviews, technology introductions and in vitro studies, only three full text articles were identified. After searching for related articles, four additional studies were included that compared reciprocal and rotary instruments [27–33]. However, Relvas et al. used a verbal rating score rather VAS to evaluate PP [30]; Nekoofar et al. [32] reported only the mean VAS score, rather than PP morbidity; and Shahi et al. [29] reported rate of PP following treatment with two different rotary instruments. Kherlakian et al. [28] contacted patients by phone while the VAS scale should be administered in written form [34]; These four studies were excluded. Three studies were included in the final meta-analysis [27, 28, 31, 33] (Fig. 1) (Table 1). Risk of bias assessment indicated a low risk for all included randomized clinical trials (Table 2). Two studies [27, 28, 33] used similar analgesics (400 mg ibuprofen) while one study [31] did not clarify the analgesics used.

**Table 1:** Studies included

Study	Centers	Reciprocating vs rotary machine type			Patients included (n)			Visit	Follow up (days)
		RECIPROC	Wave One	Rotary	RECIPROC	Wave One	Rotary		
Gambarini et al. 2013 [33]	1	/	Wave One	TF	/	30	30	Single	3
Neelakantan et al. 2015 [27]	2	RECIPROC	/	One Shape	605	/	605	Single	7
Pasqualini et al. 2015 [31]	1	/	Wave One	Pro Taper		24	23	Single	7

**Table 2:** Risk of bias assessment for included RCTs

Author	Random sequence generation	Allocation concealment	Blinding of participants and personnel	Blinding of outcome	Incomplete outcome data	Selective reporting	Other sources of bias	Overall risk of bias
Gambarini et al. 2013 [33]	Low	Unclear	Low	Low	Low	Low	Low	Low
Neelakantan et al. 2015 [27]	Low	Low	Low	Unclear	Low	Low	Low	Low
Pasqualini et al. 2015 [31]	Unclear	Unclear	Low	Low	Low	Low	Low	Low

**Fig. 2:**
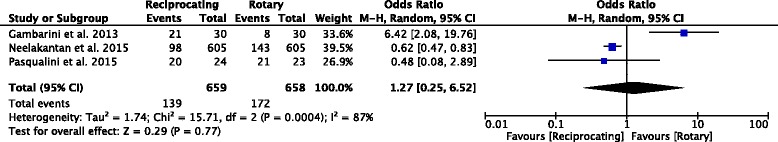
Post endodontic pain incidence odds ratio comparing reciprocating with rotary instruments. There were 1,317 patients included in the whole study and odds ratio was 1.27 favored rotary instruments in the PP incidence for single visit canal therapy patients

The included trials involved a total of 1,317 patients, 659 treated with reciprocating instruments and 658 treated with rotary instruments. PP was reported in139 patients (21%) in the reciprocating group and 172 (26%) in the rotary group. The Tau^2^ was 1.74, Chi^2^ was 15.71, I^2^ = 87%, Z = 0.29 (*P* = 0.77), and Odds ratio was 1.27 (0.25, 6.52) (Fig. 2).

**Fig. 3:**
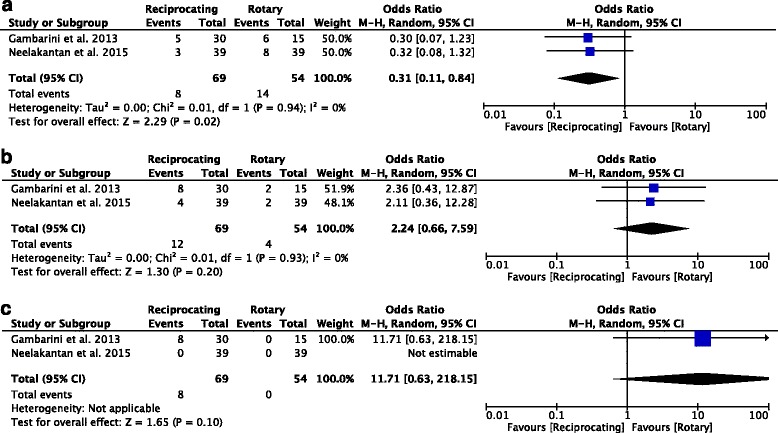
Subgroups analysis of mild (**a**), moderate (**b**), severe (**c**) levels PP incidence odds ratios was 0.31 (0.11, 0.84), 2.24 (0.66, 7.59) and 11.71 (0.63, 218.15) respectively comparing reciprocating with rotary instruments

One study [31] was excluded from subgroup analysis as no pain classification was included, while in the remaining studies the incidence odds ratios of mild, moderate, and severe PP were 0.31 (0.11, 0.84), 2.24 (0.66, 7.59) and 11.71 (0.63,218.15), respectively (Fig. 3).

**Fig. 4:**
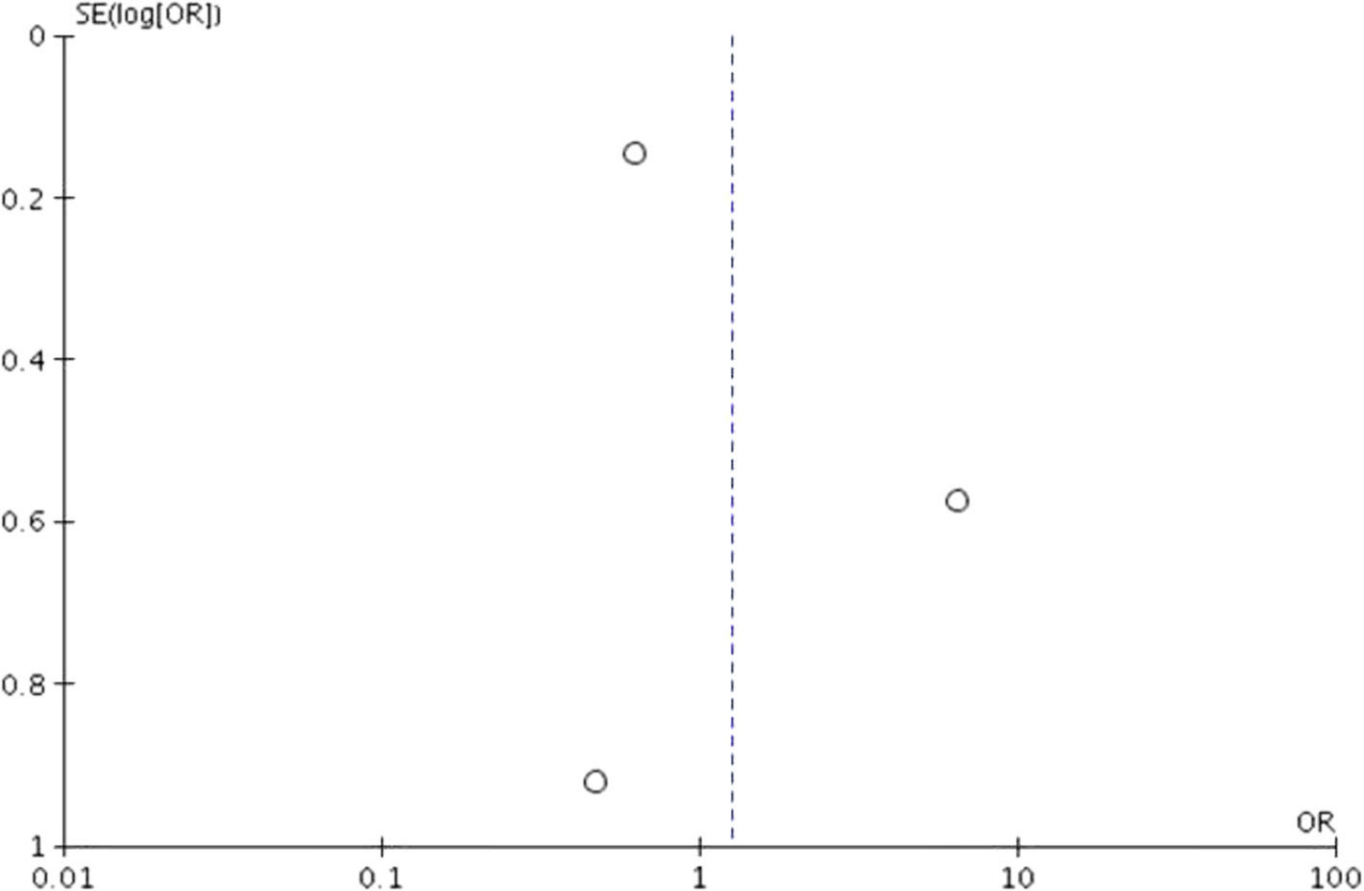
Funnel plot showed that no publication bias was found in the included four studies

Funnel plot analysis indicated no publication bias among studies (Fig. 4).

## Discussion

In this meta-analysis, the rate of PP following canal preparation using either reciprocating or rotary instrument was assessed. The PP incidence odds ratio was 1.27, favoring rotary instruments. Subgroup analysis of pain levels indicated that mild PP incidence favored reciprocating instruments while moderate and severe PP incidence favored rotary instruments.

Clearly, the incidence of PP was lower in patients treated with rotary instruments than reciprocating instruments, perhaps because rotary instruments reducedebris extrusion, which decreases the irritation and minimizes inflammation and the release of chemical substances [34]. The released mediators such as neuropeptides, arachidonic acid metabolites, cytokines, lysosomal enzymes, platelet-activating factor, fibrinolytic peptides, vasoactive amines, anaphylatoxins and kinins, might lead to postoperative complications [34]. Furthermore, Nair et al. [35] and Cavidedes-Bucheli et al. [36] showed the use of different instrumentation techniques could result in different amount of extruded debris and neuropeptides, which may potentially explain the observed differences in PP severity. Furthermore, De Deus et al. [37] compared a full range of Pro Taper Universal instruments in rotary motion with reciprocating motion in 54 patients, and reported that the percentage of residual pulp tissue was similar in round canals, while significantly less with rotary motions. At the same time, the advantages of reciprocating motion should also be emphasized: root canal retreatment was faster when reciprocating motion was used [38], and equally effective to rotary motion [39]. Our results suggest that rotary instruments yield lower overall PP incidence than reciprocating instruments in single visit canal preparation patients.

Numerous canal instrument systems have been developed, but all exhibit some degree of debris extrusion despite differences in design, cross-sectional configuration, and application methods [20, 40]. Careful control of working length might reduce the extrusion of material through the apical foramen, but cannot prevent it completely [6]. Rotary instruments have been developed with symmetrical and asymmetrical rotary motion [41]. The center of asymmetrical rotary instruments is positioned off-center relative to the instrument’s central axis of rotation. During rotation, a mechanical wave of motion travels along the length of the working part of the instrument and minimizes contact between the file and dentin [28]. In this case, rotary systems could yield cleaner canals with less debris accumulation than reciprocating instruments [42]. Previously, the reciprocating motion involves an initial rotation in a counterclockwise direction, which allows the instrument to penetrate and cut the dentin. Thereafter follows a rotation in the opposite direction, which allows the instrument to be released [28, 43]. Recently, use of a unique, proprietary movement, combining reciprocation and continuous rotation (TF Adaptive, Sybron Endo, USA) [33] was reported to not significantly improve PP condition when compared to a rotary crown-down technique using TF instruments and a reciprocating single-file technique using Wave One instruments. However, the small number of included patients in that trial limited its statistical power. Moreover, the fact that reciprocating instruments led to more debris is not related only to the kinematics, but also to the irrigation protocol used [44].

The level of debris extrusion in canal preparation is reported to vary widely between different mechanical systems [19, 45]. In vitro studies have shown that reciprocating systems can cause greater debris extrusion [40], or accumulation of debris in the root canal [42] than rotary systems, possibly as a result of the reverse motion of the reciprocating instrument. On the contrary, another in vitro study reported that less apical extrusion of bacteria was produced using the reciprocating system [46]. However, results generated in vitro may not apply to clinical cases.

Interestingly, subgroup analysis for pain degree indicated that the incidence of mild PP was higher in patients treated with reciprocating instruments, while the incidence of moderate and severe PP was lower in patients treated with rotary instruments. This could be explained by the different study and instrument design. Studies included in this meta-analysis varied in terms of cross section, cutting-edge design, taper, tip type, configuration, use concept, flexibility, alloy type, number of files used, kinematics, and cutting efficacy. Further studies controlling for these variables will be required to clarify the incidence, degree and duration of PP following canal therapy.

The limitations of this study lies in limitations typical of meta-analyses: first, homogeneity of the patients involved, inconsistent instrumentation protocol and so on; second, different file size and taper were applied in the included studies, while subgroup analysis of different file size and taper were impossible as PP incidence was not accordingly reported; third, PP was evaluated at different time points, although 1 week follow-up was the most common; forth, the VAS used to assess pain is subjective, rather than objective. Furthermore, Gambarini et al. acquired VAS using an independent evaluator without knowledge of visit group under examination [33], while VAS must be used without an evaluator interference; fifth, analysis of the frequency and dose of analgesic medication may also have provided additional information, but pooling this data was difficult. Finally, all included studies involved only patients treated at a single visit, so we cannot extrapolate the results to patients treated over multiple-visits. Future studies should consider and avoid these limitations.

## Conclusion

This meta-analysis indicates that the use of rotary instruments in canal preparation is associated with a lower incidence of post-endodontic pain than reciprocating instruments.

## Abbreviations

CI: Confidence interval
NiTi: Nickel-titanium
OR: Odds ratio
PP: Post-endodontic pain
VAS: Visual Analogue Scale
